# Serum Growth Factors in Schizophrenia Patients

**DOI:** 10.3390/cimb45040215

**Published:** 2023-04-07

**Authors:** Anastasiia S. Boiko, Irina A. Mednova, Elena G. Kornetova, Nikolay A. Bokhan, Svetlana A. Ivanova

**Affiliations:** 1 Mental Health Research Institute, Tomsk National Research Medical Center of the Russian Academy of Sciences, 634014 Tomsk, Russia; 2 University Hospital, Siberian State Medical University, 634050 Tomsk, Russia; 3 Department of Psychiatry, Addictology and Psychotherapy, Siberian State Medical University, 634050 Tomsk, Russia

**Keywords:** growth factors, schizophrenia, biomarkers, clinical features, EGF, VEGF, FGF-2, TGF-α, PDGF

## Abstract

Some hypotheses include schizophrenia as a neurodevelopmental disorder, which indicates a special role in growth factors and neuroglia in the development of schizophrenia symptoms. Growth factors are cytokine molecules that play an important role in the regulation of tissue nucleation, cell development, survival, and migration of all tissues in organisms, including the brain and nervous system. The aim of the study was to determine the serum concentration of six growth factors (EGF, VEGF, FGF-2, TGF-α, PDGF-AA, PDGF-AB/BB) in schizophrenia patients and to identify the correlations with clinical characteristics. After signing an informed consent form, 236 schizophrenia patients (F20 according to the ICD-10) and 102 healthy people were recruited in the study. In patients with schizophrenia, we observed a significant elevation in the TGF-α and PDGF-AA serum levels. The duration of schizophrenia was significantly positively correlated with the FGF-2 level. The PANSS total score had a positive correlation with the FGF-2 level and a negative correlation with the TGF-α level. Our results and literature indicate the involvement of growth factors in the mechanisms of development of schizophrenia. Combined biomarker screening seems to be necessary to improve diagnosis and clinical follow-up of patients with severe mental illnesses.

## 1. Introduction

Schizophrenia is considered one of the most serious mental illnesses. Many patients cannot fully recover, and even among those who receive positive therapeutic results, the diagnosis has fateful aftermaths, including stigmatization, social isolation and loneliness in their personal lives [1]. This mental disorder is characterized by a complex of positive symptoms (hallucinations, disorganized speech and delusions), negative symptoms (reduction in motivation and weak expressiveness), and cognitive deficits (disturbed executive functions, decrease in memory and reduced speed of mental processing) [2].

For many years, the concept of immune dysfunction in the pathogenesis of schizophrenia was discussed and developed based mainly on changes in immune markers in peripheral blood [3,4]. Interest in this field of research has been revived in the last decade, and the schizophrenia neuroimmune hypothesis has been re-strengthened, supported by both early studies and modern data [5,6,7,8]. Among the extensive cytokine profile, a separate group of growth factors can be distinguished.

On the other hand, some researchers hypothesize that schizophrenia is a neurodevelopmental disorder; thus, growth factors and neuroglial cells are important molecules mediating the development of schizophrenia symptoms. Growth factors are cytokine molecules that play an important role in the regulation of tissue nucleation, cell development, survival and migration of all tissues in organisms, including the brain and nervous system [9]. Neurons and neuroglia have a close anatomical and functional relationship. One of the functions of astrocytes in the central nervous system (CNS) is the expression of receptors for various growth and immune factors as well as neurotransmitters; in turn, neurons can control growth factors that function through a common set of signaling molecules and intracellular transduction pathways [10]. In the CNS, growth-factor-mediated glial–neuronal relationships play a critical role during neural pathway development; they also play a critical role in the maintenance of normal adult brain function and neuroprotection [11].

The epidermal growth factor (EGF) family, such as EGF itself, transforming growth factor alpha (TGF-α), amphiregulin, heparin-binding EGF, betacellulin, and epiregulin, are polypeptides that vary in the fact that their soluble forms are proteolytically derived from their integral membrane precursors [11]. Few studies have shown that EGF and TGF-α are expressed at very high levels in the central nervous system compared to other family members due to their much higher content and wider distribution [12].

EGF is a signaling molecule with a variety of functions, one of which is the production of oligodendrocytes in the adult cerebrum [13]. In brain tissues, including neurons and astrocytes, EGF is widely expressed and promotes the proliferation, differentiation, migration and survival of these cell precursors [14]. There is information that cognitive impairment and some negative symptoms of schizophrenia are associated with dysfunction in the prefrontal cortex [9]. Some studies found that the forebrains of schizophrenia patients show decreased expression of EGF in the prefrontal cortex as well as in the striatum [15]. A low concentration of EGF is also observed in the blood of psychiatric patients [16].

TGF-α is a ligand for the EGF receptor and is involved in neurogenesis [12]. It has been assumed that neurogenesis deficiency can play an important role in the development of some serious mental disorders, for example, depression [17]. TGF-α has also been considered as a biomarker for cocaine abuse [18].

Vascular endothelial growth factor (VEGF) is a signaling molecule that stimulates vasculo- and angiogenesis [19]. It is also a powerful mitogen for endothelial types of cells and astrocytes [20]. VEGF functions are mediated by their specific receptors expressed by the endothelium, astrocytes, and neuronal cells of any degree of maturity [21]. VEGF contributes to the neuroprotection and cell regeneration of the CNS and also stimulates axon growth and neuronal differentiation [22]. Therefore, VEGF may promote the regeneration of neuronal cells after brain injury, as well as neurogenesis in the adult brain [23].

Fibroblast growth factor (FGF) is a protein superfamily consisting of 18 protein species with four homologous molecule types that bind to four surface tyrosine kinase receptors. In the adult brain, these proteins control neurogenesis, gliogenesis, axonal outgrowth, myelination, and memory formation [9]. Studies have shown the involvement of the FGF family in adult neurogenesis [24,25], which may indicate the involvement of the FGF in neuroplasticity processes, which plays a neuromodulatory role in how the organism reacts to the environment and copes with it. FGF-2 (also known as FGF-beta or bFGF) also enhances the proliferation and differentiation of dopaminergic neurons [26], which is very important in schizophrenia [27]. The expression of FGF-2 in peripheral blood leukocytes of schizophrenics was different from that of healthy controls [28].

Human-platelet-derived growth factor (PDGF) is a potent mitogen for diploid fibroblasts, osteoblasts, vascular smooth muscle cells, and brain glial cells. It is one of the main mitogens in human serum, formed from platelets [29]. The PDGF family comprises four members (-A, -B, -C and -D), which are assembled from disulfide-linked homo- or hetero-dimers of two different but related chains. Additional intrachain disulfide bonds are required for proper folding of the PDGF protein [30]. PDGF has many biological effects on target cells. At the first stage, PDGF interacts with specific receptors of cell membranes and induces a tyrosine-specific kinase capable of autophosphorylation of the receptor and phosphorylation of the cell membrane and cell protein at tyrosine residues [31]. In addition to its mitogenic properties, PDGF contributes to the stimulation of some important cellular metabolic processes, including the synthesis of proteins, lipids, and prostaglandins [32]. It is evidently an important developmental factor and takes part in the processes of tissue regeneration and remodeling.

The aim of our research was to determine the concentration of serum growth factors in schizophrenia patients and to identify the correlations with clinical characteristics.

## 2. Materials and Methods

### 2.1. Participants

The research was conducted in accordance with the principles of the Declaration of Helsinki of the World Medical Association (1975, revised in Fortaleza, Brazil, 2013). The study protocol and conducted research were approved by the Bioethical Committee of the Mental Health Research Institute, Tomsk National Research Medical Center of the Russian Academy of Sciences (Tomsk NRMC) (approved on 24 April 2018, No. 187). A total of 236 patients with schizophrenia (F20 according to the International Classification of Diseases-10 (ICD-10) and 102 mentally and somatically healthy people who gave written informed consent were included in the study. The inclusion criteria for the study were age 18–65 years and no signs of acute and chronic infectious–inflammatory and autoimmune diseases. In this study, we did not recruit any individuals with comorbid neurological and somatic diseases that would have made it difficult to objectively assess their clinical condition, or persons who used psychoactive substances.

We conducted psychometric determinations of the severity of schizophrenia symptoms using a Positive and Negative Syndromes Scale (PANSS) [33]. This instrument consists of a 7-item scale measuring the severity of positive symptoms of schizophrenia on a 1–7 point Likert scale, a 7-item scale that does the same with negative symptoms, and a 16-item scale for general psychopathological symptoms (such as tension, anxiety, disorientation, etc.).

F20.x1-3 in accordance with the ICD-10 was determined as an episodic clinical course of schizophrenia and F20.x0 was determined as a continuous clinical course of schizophrenia. The prescribed doses of antipsychotics were converted into the chlorpromazine equivalent (CPZeq) [34].

### 2.2. Laboratory Examination

Blood samples were taken from the antecubital vein of the participants early in the morning, on an empty stomach (more than 8 h of fasting), into tubes with a clot activator. Serum was obtained by a standard method (centrifugation of whole blood for 30 min at 2000× *g* at +4 °C), after which the samples were frozen and stored at −80 °C. Patients were examined in the first days after admission to the hospital.

The concentration of six growth factors (EGF, VEGF, FGF-2, TGF-α, PDGF-AA, PDGF-AB/BB) was determined in blood serum by a panel HCYTMAG-60K-PX41, MILLIPLEX MAP (Merck, Darmstadt, Germany) using xMAP technology on multiplex analyzers Magpix and Luminex 200 (Luminex corp., Austin, TX, USA) (instruments are based at the Core Facility “Medical Genomics,” Tomsk National Research Medical Center). This technology determines the concentration of a large number of analytes simultaneously in a small volume of a biological sample. Growth factor concentrations were measured in pg/mL, followed by data exported to the xPONENT software (Luminex, Austin, TX, USA) and primary conversion with the software Milliplex Analyst (Merck, Darmstadt, Germany).

### 2.3. Statistical Analysis

Statistical data processing was carried out in the SPSS software (v.20, for Windows) and R software package (v. 4.0.4) using standard functions and specialized packages.

Quantitative data were checked for compliance with the normal distribution law. The results are presented as median (Me) and quartiles (Q) (lower/first and upper/third). We used the non-parametric Mann–Whitney U-test to detect statistically significant differences between quantitative variables and the Chi-square test to evaluate statistically significant differences between qualitative features. We used Spearman’s correlation analysis to identify relationships between quantitative traits. The critical significance level was established at less than 0.05.

## 3. Results

### 3.1. The Main Demographic and Clinical Characteristics of Participants

For this study, we recruited 236 patients with a verified diagnosis of schizophrenia (F20 according to ICD-10) who were treated in Siberia psychiatric hospitals. Patients were hospitalized due to an exacerbation in the continuous course or relapse in the episodic course, while the average PANSS total score was 100 [87; 109]. The age of the patients was 36 [30; 46] years (from 18 to 68). The distribution of men and women was equal in the main group (48.7% and 51.3% respectively) and in the healthy persons group. Schizophrenia manifested at 24.5 [21; 32] years and the duration of the disorder was 12 [5; 19] years. The comparison group consisted of 102 mentally and physically healthy people at the time of inclusion in the study who did not have a history of psychiatric or severe somatic diseases. Both groups were comparable in terms of the listed demographic characteristics, as evidenced by the lack of statistical significance between them. The main socio-demographic and clinical parameters of participants are presented in Table 1.

### 3.2. The Concentration of Growth Factors in Patients with Schizophrenia

The concentrations of growth factors in the patients’ and healthy people’s serum are presented in Table 2.

Statistically significant differences in the concentrations of two growth factors were found in patients with schizophrenia compared to healthy persons. The level of transforming growth factor alpha in the patient group was 5.06 [3.98; 7.04] pg/mL, which exceeds its level in the healthy control group (4.61 [2.81; 7.2] pg/mL) (*p* = 0.013). A significant difference in the level of platelet-derived growth factor (subunit AA) was observed in schizophrenia patients (3320 [2281; 4392] pg/mL) compared with healthy people (853.99 [1555; 4177] pg/mL, *p* = 0.04).

The study subjects were evaluated for gender difference based on gender: 47 men (46%) and 55 women (54%) in the control group; 115 men (49%) and 121 women (51%) in the schizophrenia group.

Healthy men had a higher level of VEGF (178.21 [114.64; 308.87] pg/mL) compared with healthy women (130.41 [83.74; 199.13] pg/mL) (*p* = 0.015). Other growth factors did not differ based on sex in the control group. A decreased EGF level was observed in female patients with schizophrenia (183.51 [129.66; 266.76] pg/mL) compared to male patients (158.76 [105.25; 228.23] pg/mL) (*p* = 0.03). Other serum growth factors were similar in male and female patients.

Comparative analysis of the concentration of growth factors was carried out separately among men (Table 3) and then among women (Table 4), depending on the presence of a mental disorder.

A significantly lower level of VEGF was found in the blood serum of men with schizophrenia compared with healthy men.

Women from the control group and the group with schizophrenia had differences in the levels of two growth factors, similar in the general groups of healthy people and patients, but even more highly statistically significant. Higher levels of TGF-α and PDGF-AA were observed in the blood serum of female patients with schizophrenia compared with the concentrations in mentally healthy women (*p* = 0.006 and *p* = 0.025, respectively).

### 3.3. The Concentration of Growth Factors on the Clinical Features of Schizophrenia

Further, the levels of the studied growth factors were analyzed depending on the clinical parameters of schizophrenia.

Depending on the leading schizophrenia symptoms, patients were separated into two groups: 118 patients with leading negative symptoms and 117 patients with leading positive symptoms. The concentration of growth factors did not differ in the compared groups (Table 5).

The course of schizophrenia is divided into episodic and continuous according to ICD-10 (F20.x1-3 for episodic clinical course and F20.x0 for continuous clinical course). In the study group, 94 patients were identified with an episodic course and 102 patients were identified with a continuous course. Our study identified 40 patients with a follow-up period of less than a year, which does not allow clinicians to determine the type of disease course at this stage. We found no significant differences when comparing serum growth factor concentrations in episodic and continuous schizophrenia (Table 6).

The obtained results led us to a follow-up evaluation of the data, which included the identification of relationships between growth factors and quantitative characteristics such as age, duration of illness, and PANSS scores.

### 3.4. Spearman’s Correlation Analysis

The results of Spearman’s correlation analysis between pairs of variables: serum growth factors, age, duration of disease, and PANSS scores in a group of schizophrenia patients are shown in Table 7.

Interestingly, a significant positive correlation was observed between FGF-2 and schizophrenia duration (Po = 0.157, *p* = 0.016), while other growth factors did not show an association with this characteristic. The age of the patients correlated positively with VEGF and FGF-2 and negatively with EGF and PDGF-AB/BB. The PANSS total score had a positive correlation with FGF-2 and a negative correlation with TGF-α. Additionally, serum growth factors showed several weak and medium positive and negative correlations among themselves. EGF correlated positively with TGF-α and platelet-derived growth factors and negatively with FGF-2. VEGF correlated with FGF-2. TGF-α also correlated with PDGF-AB/BB. Additionally, platelet-derived growth factors correlated with each other.

## 4. Discussion

Even minor changes in the structure, expression and/or function of growth factors can alter the development of the brain and nervous system. Changes in the levels of growth factors may be related to the pathogenesis and associated with the clinical manifestations of a number of mental illnesses [9,15,35,36]. In the past, several growth factors have been explored in schizophrenia, and the results indicate that these signal molecules play an important role in the pathogenesis and development of the clinical symptoms of this disease [37,38,39].

In the current study, we found significantly higher levels of transforming growth factor alpha and platelet-derived growth factor (subunit AA) in patients with schizophrenia compared with healthy individuals. PDGF changes may be associated with the development of cognitive impairment characteristic of schizophrenia, due to their possible pathogenesis associated with vascular disturbances [40]. In line with this, laboratory studies in PDGF receptor knockout mice showed a decrease in the number of neuronal cells and the development of cognitive deficits [30].

It is assumed that since TGF and its related molecules are synthesized by neuronal and glial cells and regulate glial and neuronal functions, they can be considered as a mechanism for relating neuroglia and neurons to the environment [11]. Altered patterns of TGF and EGF expression were involved in neuronal–glial interactions in several neuropsychiatric illnesses and also changed in response to damage to the nervous tissue [12].

The most pronounced differences in the concentration of these growth factors (TGF-α and PDGF-AA) were observed in female patients with schizophrenia.

According to the literature, growth factors contribute to the development of tissues as well as the emergence and migration of cells and provide their survival in all tissues of the body, including the brain and the CNS [9]. According to one hypothesis, schizophrenia is a neurodevelopmental disease [1,3,5]. Given this, an increase in the concentration of the TGF-α and PDGF can be considered a compensatory reaction in response to damage and death of nerve cells, which can increase in schizophrenia.

Plasma VEGF levels were significantly lower in antipsychotic-naive patients with schizophrenia compared to healthy subjects [21]. Decreased VEGF expression in the dorsolateral prefrontal cortex was found in schizophrenia patients [41]. VEGF plays an important role in the protection and regeneration of CNS cells and stimulates the growth of axons and differentiation of neurons [22,42]. We also found a decrease in the serum level of this growth factor in male patients suffering from schizophrenia. Taking into account the available literature and the obtained results, we can assume an increase in the processes of nerve destruction and a weakening of neuron regeneration in schizophrenia, as evidenced by a decrease in VEGF.

FGF is a large family of peptides, with FGF-2 of great biological importance as an inducer of fibrogenesis. FGF is an unsecreted growth factor released only as a result of cell death or damage [43]. This links FGF to the effects of glutamatergic neurotoxicity and neuroinflammation that we have also previously investigated [44,45]. In our study, we found a significant correlation between this growth factor and the duration of mental disorder. This may indicate an increase in destructive processes, which worsens patients’ quality of life and subsequent prognosis.

Our results contribute to the growing evidence for the involvement of growth factors in the pathophysiology of mental illness, including schizophrenia [9]. It is still unclear how and in what anatomical structures they exert this action. For some aspects of schizophrenia, this could be in the dorsal diencephalic connection system (DDCS) via the habenula [46,47]. Precisely like some other researchers [48], we would like to consider the growth factors as potential biomarkers for the diagnosis and/or prognosis of certain a psychiatric disease [8]. Currently, there is no specific serum biomarker for each different mental disorder, but combined screening of biomarkers whether or not in relation to specific stimulation of the DDCS [47] seems to be necessary to improve diagnosis and clinical follow-up of patients with psychiatric disease.

An important limitation of our study is the inability to assess the effect of antipsychotic therapy on growth factor concentrations due to the long duration of the illness in many patients and the therapy with various classes of antipsychotics in previous exacerbations of psychosis or maintenance doses until the present hospitalization.

Thus, our pilot studies, for the first time, showed changes in the TGF-α and PDGF-AA serum levels in schizophrenia patients (especially in female patients) and VEGF in male patients in comparison with healthy persons and an increase in FGF-2 in the long course of the disease. However, further studies are needed for evaluation involving the growth factors in schizophrenia pathogenesis.

## Figures and Tables

**Table 1:** Basic demographic and clinical characteristics of participants.

Parameter		Healthy Persons (*n* = 102)	Patients (*n* = 236)	*p*-Value
Age, years		35 [27; 47]	36 [30; 46]	0.477
Gender, *n* (%)	Male	47 (46.1%)	115 (48.7%)	0.654
	Female	55 (53.9%)	121 (51.3%)	
Age of manifestation, years		-	24.5 [21; 32]	
Duration of disorder, years		-	12 [5; 19]	
Clinical course, *n* (%)	Episodic course	-	94 (43.1%)	
	Continuous course	-	102 (46.8%)	
PANSS, positive symptoms Score		-	21 [17; 25]	
PANSS, negative symptoms Score		-	25 [21; 28]	
PANSS, general psychopathology Score		-	53 [44; 58]	
PANSS, total score		-	100 [87; 109]	
Leading symptomatology, *n* (%)	Negative	-	118 (50.2%)	
	Positive	-	117 (49.8%)	
Duration of antipsychotic treatment, years		-	7 [3.1; 14.9]	
Total CPZeq		-	342.5 [200.1; 676]	

Note: Data are presented as median [lower quartile; upper quartile]; PANSS: Positive and Negative Syndrome Scale; CPZeq: chlorpromazine equivalent. Comparisons between groups were performed using the chi-square test for gender distribution and the Mann–Whitney U-test for age.

**Table 2:** Concentration of serum growth factors in serum of patients with schizophrenia and healthy persons (Me [Q1; Q3]).

Growth Factors, pg/mL	Healthy Persons (*n* = 102)	Patients with Schizophrenia (*n* = 236)	*p*-Value
EGF	146.8 [74.83; 259.72]	172 [116.1; 240.43]	0.121
VEGF	146.98 [108.44; 252.66]	133.68 [82.78; 194]	0.119
FGF-2	65.11 [46.61; 132.72]	67.87 [43.15; 139.96]	0.75
TGF-α	4.61 [2.81; 7.2]	5.06 [3.98; 7.04]	0.013 *
PDGF-AA	2853.99 [1555; 4177]	3320 [2281; 4392]	0.04 *
PDGF-AB/BB	12263 [8197.75; 23576.57]	11100 [8637; 19878]	0.119

Note: Data are presented as median [lower quartile; upper quartile]; comparisons between groups were performed using the Mann–Whitney U-test. *—significant *p*-value < 0.05.

**Table 3:** Concentration of serum growth factors in male patients with schizophrenia and healthy persons (Me [Q1; Q3]).

Growth Factors, pg/mL	Healthy Men (*n* = 47)	Men with Schizophrenia (*n* = 115)	*p*-Value
EGF	152.37 [85.03; 288.14]	183.51 [129.66; 266.76]	0.294
VEGF	178.21 [114.64; 308.87]	141.7 [82.78; 222.48]	0.029 *
FGF-2	71.2 [46.61; 137.6]	59.16 [42.36; 137.6]	0.419
TGF-α	4.7 [3; 8.07]	4.96 [3.74; 7.08]	0.456
PDGF-AA	3174.55 [1554.25; 4866.72]	3320 [1940.5; 4531]	0.56
PDGF-AB/BB	12258 [8688; 18024]	11257 [8955.5; 25078.13]	0.855

Note: Data are presented as median [lower quartile; upper quartile]; comparisons between groups were performed using the Mann–Whitney U-test. *—significant *p*-value < 0.05.

**Table 4:** Concentration of serum growth factors in female patients with schizophrenia and healthy women (Me [Q1; Q3]).

Growth Factors, pg/mL	Healthy Women (*n* = 55)	Women with Schizophrenia (*n* = 121)	*p*-Value
EGF	139.77 [72.12; 252.42]	158.76 [105.25; 228.23]	0.269
VEGF	130.41 [83.74; 199.13]	130.95 [83.67; 177.18]	0.933
FGF-2	54.15 [45.23; 104.41]	85.78 [43.17; 140.54]	0.189
TGF-α	4 [2.46; 6.14]	5.15 [4.07; 7.01]	0.006 *
PDGF-AA	2459 [1557.27; 4038.27]	3317 [2658.25; 4259.25]	0.025 *
PDGF-AB/BB	12274.05 [7419.3; 26344.14]	11032 [8296; 1823]	0.369

Note: Data are presented as median [lower quartile; upper quartile]; comparisons between groups were performed using the Mann–Whitney U-test. *—significant *p*-value < 0.05.

**Table 5:** Concentration of serum growth factors in patients with leading negative or positive symptoms of schizophrenia (Me [Q1; Q3]).

Growth Factors, pg/mL	Negative Symptomatic (*n* = 118)	Positive Symptomatic (*n* = 117)	*p*-Value
EGF	176.35 [119.07; 234.53]	165.73 [110.78; 258.51]	0.969
VEGF	130.95 [82.78; 195.46]	133.68 [80.58; 193.62]	0.947
FGF-2	64.73 [41.07; 139.96]	68.77 [43.99; 140.54]	0.407
TGF-α	4.75 [3.55; 6.99]	5.27 [4.1; 7.36]	0.124
PDGF-AA	3322 [1905.25; 4482.25]	3308 [2508.75; 4304.75]	0.788
PDGF-AB/BB	10970.5 [8225.75; 24894.34]	11323 [8898; 18905.5]	0.635

Note: Data are presented as median [lower quartile; upper quartile]; comparisons between groups were performed using the Mann–Whitney U-test.

**Table 6:** Concentration of serum growth factors in patients with episodic or continuous clinical course of schizophrenia (Me [Q1; Q3]).

Growth Factors, pg/mL	Episodic Course (*n* = 94)	Continuous Course (*n* = 102)	*p*-Value
EGF	176.35 [119.07; 234.53]	165.73 [110.78; 258.51]	0.997
VEGF	130.95 [82.78; 195.46]	133.68 [80.58; 193.62]	0.528
FGF-2	64.73 [41.07; 139.96]	68.77 [43.99; 140.54]	0.286
TGF-α	4.75 [3.55; 6.99]	5.27 [4.1; 7.36]	0.172
PDGF-AA	3322 [1905.25; 4482.25]	3308 [2508.75; 4304.75]	0.456
PDGF-AB/BB	10970.5 [8225.75; 24894.34]	11323 [8898; 18905.5]	0.189

Note: Data are presented as median [lower quartile; upper quartile]; comparisons between groups were performed using the Mann–Whitney U-test.

**Table 7:** Spearman’s correlation analysis between growth factors, age, duration of illness and PANSS score in schizophrenia patients.

Parameter	Po, *p*-Value	EGF	VEGF	FGF-2	TGF-α	PDGF-AA	PDGF-AB/BB
Age	Po *p*-value	−0.131 * 0.045	0.133 * 0.044	0.246 * <0.001	−0.079 0.23	−0.029 0.669	−0.133 * 0.042
Duration of illness	Po *p*-value	−0.049 0.456	0.051 0.442	0.157 * 0.016	−0.075 0.254	−0.004 0.957	−0.121 0.064
PANSS (total score)	Po *p*-value	−0.066 0.315	0.037 0.584	0.172* 0.009	−0.212 * 0.001	−0.066 0.337	−0.079 0.232
EGF	Po *p*-value	1	−0.012 0.859	−0.196 * 0.003	0.291 * <0.001	0.229* 0.001	0.292 * <0.001
VEGF	Po *p*-value		1	0.365 * <0.001	0.105 0.114	−0.015 0.823	0.074 0.265
FGF-2	Po *p*-value			1	−0.046 0.483	−0.115 0.09	−0.127 0.051
TGF-α	Po *p*-value				1	0.13 0.056	0.278 * <0.001
PDGF-AA	Po *p*-value					1	0.253 * <0.001
PDGF-AB/BB	Po *p*-value						1

Note: Po—Spearman’s coefficient. * *p*-value—significance of differences < 0.05.

## Data Availability

The datasets are available on reasonable request to Prof Svetlana A. Ivanova (ivanovaniipz@gmail.com), following approval of the Board of Directors of the MHRI, in line with local guidelines and regulations.
